# Psychometric Properties of the Original and Short Form of the Inventory of Callous-Unemotional Traits in Detained Female Adolescents

**DOI:** 10.1007/s10578-015-0601-8

**Published:** 2015-10-22

**Authors:** Olivier F. Colins, Henrik Andershed, Samuel W. Hawes, Patricia Bijttebier, Dustin A. Pardini

**Affiliations:** 1 Department of Child and Adolescent Psychiatry, Curium-Leiden University Medical Center, Endegeesterstraatweg 27, 2342 AK Oegstgeest, Leiden, The Netherlands; 2 School of Law, Psychiatry, and Social Work, Örebro University, Örebro, Sweden; 3 Department of Psychiatry, University of Pittsburgh Medical Center, Pittsburgh, PA USA; 4 Faculty of Psychology and Educational Sciences, KU Leuven, Leuven, Belgium

**Keywords:** Callous, Forensic, Female delinquents, Psychometric, Antisocial

## Abstract

This study examines the psychometric properties of the self-report version of the Inventory of Callous-Unemotional Traits in 191 detained female adolescents (*M* = 15.76, *SD* = 1.02). Evidence supporting the validity of the ICU scores was generally weak, largely due to poor functioning of the Unemotional subscale. Results from confirmatory factor analyses demonstrated support for a recently proposed shortened version of the ICU consisting of two subscales (Callousness and Uncaring). Both subscales showed acceptable to good internal consistency. This short-form version also improved criterion validity, though some issues regarding its convergent validity need further consideration. In conclusion, this study suggests that a short-form version of the ICU that includes a subset of the original items may hold promise as an efficient and valid method for assessing CU traits.

## Introduction

Callous-Unemotional (CU) traits in youth are similar to the affective features of adult psychopathy and are commonly characterized by deficient empathy and guilt, insensitivity to others’ feelings, and shallow emotions. A large body of studies have found that CU traits in children and adolescents are positively associated with a severe and persistent pattern of antisocial behaviour, and substance use, and negatively associated with the personality dimensions of agreeableness and conscientiousness [1]. Several tools have been used to assess CU traits in children and adolescents, though these measures often contain a relatively limited number of items that are specifically designed to assess CU traits. To comprehensively assess CU traits, the Inventory of Callous-Unemotional Traits (ICU; 2) has been developed, an important endeavour considering the presence of these traits may have implications for diagnostic classification and treatment [1].

### The Factor Structure of Inventory of Callous-Unemotional Traits

The 24-item ICU consists of both parent- and teacher-report versions for use primarily with children, as well as a self-report version for use with adolescents and young adults. Although several studies have reported that the ICU items best fit a bifactor model comprised of three factors (Callousness, Uncaring, Unemotional) and a general CU factor, the overall fit of these models is generally poor (e.g., [3, 4]), even after correlating item-residuals based on post hoc modification indices [5–7]. In addition, research has shown that the internal consistency of the Unemotional factor is often weak to marginal [4, 5, 8], and that this factor is poorly related to the other two ICU subscales (e.g., [4, 5]), antisocial behaviour (e.g., [5, 9]), and psychopathic features other than CU traits (e.g., [7, 10]). Therefore, researchers have suggested excluding some or all of the Unemotional subscale items in future revisions of the ICU measure [3, 11]. Lastly, the general factor consists primarily of significant loadings by reverse scored items, perhaps suggestive of an underlying method factor rather than a “general” CU factor [4–6].

To address these issues, a study examining the parent-report version of the ICU performed a series of item-response theory techniques to refine the measure using a clinic sample of boys (ages 6–12) exhibiting significant conduct problems [3]. This short-form ICU (SF-ICU) consisted of 12 of the original 24 ICU items, and results showed that a revised 2-factor model consisting of items tapping Callousness and Uncaring provided a good fit to the data. This revised 2-factor structure has also exhibited good model fit using the parent-report version of the ICU in sample of elementary school children [12], as has the self-report version in a community sample of young adults [3]. However, the revised 2-factor structure of the self-report ICU has not been examined in adolescent samples, and no studies have examined the factor structure of the self-report ICU among adolescent females exhibiting antisocial behavior. This is unfortunate because factor structures of tools that tap CU traits may vary across gender (e.g., [13]), or be less adequate in mixed-gender samples than in exclusively male or female samples (e.g., [14]).

### Validity of the (Short-Form) Inventory of Callous-Unemotional Traits

As cogently argued by Hopwood and colleagues [15], asserting internal structure should be regarded as just one element of construct validity among several others. Despite the lack of an appropriately fitting factor structure, studies across male and female samples have provided support for the criterion validity of the ICU by revealing significant positive correlations between ICU scores (excluding the Unemotional factor) and alternative measures of CU traits, including the Psychopathic Personality Inventory-Revised (e.g., [11]), the Childhood Psychopathy Scale (e.g., [7]), the Youth Psychopathic Traits Inventory (e.g., [10]) and the Psychopathy Checklist-Revised: Youth Version (e.g., [16]).

Psychopathic personality is defined as a constellation of co-occurring traits, including CU traits (e.g., [17]), and, therefore, positive correlations between ICU scores and psychopathic traits other than CU traits are to be expected. However, findings from research that has examined this association are quite mixed. Several studies have shown that ICU scores are significantly correlated to interpersonal and behavioral/lifestyle psychopathic traits (e.g., [16]), whereas other studies did not reveal such relations (e.g., [18]). The convergent validity of the ICU scores received further support by demonstrating the expected positive relations with oppositional defiant behavior (e.g., [18]), early onset conduct disorder (e.g., [19]), aggression (e.g., [9]), violent and non-violent offending (e.g., [4]), and substance use (e.g., [5]), and the expected negative correlations between ICU scores and the personality dimensions of agreeableness and conscientiousness (e.g., [7, 9]).

Few studies have examined the psychometric properties of the recently developed SF-ICU. Some research has shown SF-ICU scores to be negatively related to “consideration for others” [20], a finding considered to support the criterion validity of the SF-ICU. Positive relations with conduct problems, oppositional defiant problems, rule-breaking behavior and aggression [3, 12, 20] have also demonstrated evidence of convergent validity for the SF-ICU. Despite these promising initial findings, it is imperative that more in-depth validation studies are conducted.

### Validating the (Short-Form) Inventory of Callous-Unemotional Traits in Females^1^

Testing the psychometric properties of the (SF-)ICU in females samples is particularly needed, as support for this measure predominantly stems from studies that used exclusively male or gender-mixed samples. In addition, none of these studies examined gender related differences in regards to the association between (SF-)ICU-scores and other theoretically relevant variables. This is particularly unfortunate in light of evidence showing that ICU scores may be positively related to violent offending [4] and behavioral/lifestyle features of psychopathy [9] in girls, but not in boys, with the opposite association being found for reactive aggression [8]. In addition, there is also evidence that the Unemotional factor score may be negatively related to agreeableness and conscientiousness in boys, but not in girls [9]. The sole SF-ICU study that included a combined sample of boys and girls controlled for gender when studying relations between SF-ICU scores and aggression and rule-breaking behaviour, but did not present results separately for boys and girls [12].

Further examination of the psychometric properties of the (SF-)ICU in detained girls is also highly relevant in light of the CU-based *DSM*-*5* specifier for the diagnosis of conduct disorder (CD), particularly childhood-onset CD [21]. Notwithstanding the high prevalence of CD among detained girls (e.g., [22]), these girls were not included in the data analyses leading to this CU specifier [23]. Therefore, studies that support the link between (SF)ICU scores and (childhood-onset) CD are urgently needed, especially whilst relying on self-report (e.g., [23, 24]). Finally, ICU scores have been positively related to CD in young children (e.g., [19]), and higher ICU scores have been revealed in boys with an early (vs. late) onset of conduct *problems* [25]. Yet, we are aware of no (SF)-ICU study that tested if adolescents with childhood-(vs. adolescent-)onset conduct *disorder* have higher levels of CU traits.

### Current Study

The overall aim of current study is to examine the factor structure and validity of the self-report (SF-)ICU in a sample of detained female adolescents. This study aims to examine: i) the factor structure of the (SF-)ICU; ii) associations between (SF-)ICU scores and an alternative, well-validated measure of CU traits; and iii) associations between (SF-)ICU scores and theoretically meaningful constructs. The paper will substantially contribute to the literature by providing the first extensive testing of the psychometric properties of the self-report ICU and SF-ICU in detained female adolescents. Having psychometrically sound self-report measures of CU traits is extremely important when working with detained youths, as their parents are often not available or willing to provide ratings on these features (e.g., [10, 26]), and teachers are difficult to reach and often provide limited information due to high rates of school dropout and truant behaviour among detained youth [27]. Finally, this study will contribute to the literature by being the first to investigate the link between (SF-)ICU scores and a diagnosis of (childhood-onset) CD in detained female adolescents.

#### Hypotheses

With regard to the measure’s *factor structure*, we expect that only the model fit for the SF-ICU’s will be acceptable. Because the original ICU has been and is still used in many studies, we will also present descriptive statistics, reliability estimates and associations with variables of interest for the ICU. In terms of *criterion validity*, we expect all (SF-)ICU scores, other than the ICU Unemotional factor, will be positively related to the CU dimension of the Youth Psychopathic Traits Inventory (YPI; 28), a self-report tool of which the factor structure, internal consistency and validity have been supported across different settings and samples, including detained girls (e.g., [29–31]). We also expected that these (SF-)ICU scores will be positively related to the other two YPI dimensions, but that the magnitude of these correlations will be lower than correlations with the YPI CU dimension. In terms of *convergent validity*, we expected that (SF-)ICU scores (except the ICU Unemotional factor score) will also be positively associated with CD, oppositional defiant disorder (ODD), substance use disorders (SUD), aggression, and self-reported offending. In terms of *convergent validity*, we also expected that all (SF-)ICU scores will be associated with lower levels of agreeableness and conscientiousness. Based on prior work [3, 12, 20], we also hypothesized that the ICU and SF-ICU will exhibit nearly identical associations with the constructs included in the current study.

Finally, empirical work on the link between CU traits and internalizing problems produced mixed findings regardless of gender or the tool being used to assess CU traits (e.g., [30, 32]). To add to the literature on this topic, the present study will present findings from exploratory analyses examining the relation between (SF-)ICU scores and internalizing problems.

## Methods

### Participants

Participants included 191 girls residing in an all-girl Youth Detention Center (YDC) in Flanders, Belgium. Girls are referred to this YDC by a juvenile judge when charged with a criminal offense (e.g., assault, arson, theft), or because of an urgent problematic educational situation in which the girls most often display behavioural problems (e.g. truancy, running away, aggression, prostitution). Placement in this YDC represents the most severe measure allowable by a juvenile judge, and only girls demonstrating the most severe criminal and behavioural problems are assigned to this YDC. To recruit a substantial sample of detained female adolescents (younger than 18), we recruited girls during four different periods between July 2008 and December 2011 (for details see: 30). Detained girls were eligible to participate in the study if they had sufficient knowledge of Dutch and had an expected minimum detention duration of 1 month (i.e., to allow time for recruitment and interview). During these four periods a total of 272 unique girls were detained in the YDC. Of these girls, 50 did not meet inclusion criteria, 14 were not approached in time to participate in the study, 10 refused to participate, three could not be interviewed due to practical circumstances, and four did not complete the full battery of study instruments. This resulted in a total sample of 191 females (ages 12–17; *M* = 15.76, *SD* = 1.02), with 134 girls (70.2 %) of Belgian origin.

### Procedure

This study was approved by the institutional review board (IRB) of the Faculty of Psychology and Educational Sciences, Ghent University and the board of the YDC. Because screening for emotional problems is a mandatory task of the YDC, the IRB waived the requirement of parental consent. The board of the YDC agreed with this procedure. Participants were approached and assessed following a standardized protocol. Selected girls were approached individually and given oral and written information about the aims, the content, and the duration of the study. They were assured that all information provided would remain confidential and that refusal to participate would not affect their judicial status or stay in the YDC. The girls could consult their primary caregivers or other adults about participation and written informed consent was given before participation. Participating girls did not receive compensation and could ask for help when they did not understand or could not read a question.

### Measures

#### Inventory of Callous-Unemotional Traits (ICU)

The ICU [2] has 24 items that need to be scored on a 4-point Likert type scale (ranging from 0 = “not at all true” to 3 = “definitely true”). Prior work suggests that a bifactor model consisting of three subfactors (Callousness, 11 items; Uncaring, eight items; and Unemotional, five items) and one general CU factor (consisting primarily of reverse scored items) best fit the data (e.g., [4–6]). However, as noted earlier, this factor structure frequently necessitates correlating several residual items before acceptable fit is achieved. To test the 2-factor model of the SF-ICU, we used the 12 items identified by Hawes (2014). Only one of these items (i.e., “Does not show emotions”) was included in the Unemotional factor. Items from the SF-Callousness subscale tend to be negatively worded while items from the SF-Uncaring factor are positively worded and reversed scored (e.g., [3]). However, the Dutch ICU does not require reversing the score of one item due to a wording change (i.e., English version = “Is concerned about other’s feelings;” Dutch version = “Is not concerned about other’s feelings”). Therefore, this item was loaded on the SF-Callousness instead of the SF-Uncaring factor, because it appears that the positive/negative wording of items significantly influences the factor structure. ICU and SF-ICU scores are summed scores, with the total ICU and SF-ICU scores being based on 24 and 12 items, respectively.

#### Youth Psychopathic Traits Inventory (YPI)

The YPI [28] is a self-report questionnaire based on the 3-factor model of psychopathy [17]. The 50 items of the YPI are organized into ten subscales with five items in each subscale. Each YPI item is scored on a 4-point Likert type scale (ranging from 0 = “Does not apply at all” to 3 = “Applies very well”). The ten subscales form three dimensions, being a Grandiose-Manipulative dimension (GM; 20 items), the Callous-Unemotional dimension (CU; 15 items), and Impulsive-Irresponsible dimension (II; 15 items). Internal consistency for the YPI-total score in the present study was *α* = 0.92, and for the three dimensions *α* = 0.89, *α* = 0.81, and *α* = 0.87, respectively. The Dutch version of the YPI was used in the present study and summed scores were used.

#### Youth Self-Report (YSR)

The Dutch version of the YSR [33] was used as a dimensional measure of externalizing and internalizing problems. This tool consists of 118 items that are rated on a 3-point Likert type scale (ranging from 0 = “not at all true” to 2 = “often”). In this study, the following YSR subscales were used: Withdrawn/Depressed (8 items; *α* = 0.72); Anxious/Depressed (13 items; *α* = 0.87); Attention Problems (9 items; *α* = 0.75); Rule breaking Behavior (15 items; *α* = 0.82); and Aggressive Behavior (17 items; *α* = 0.87).

#### Diagnostic Interview Schedule for Children-Fourth Version (DISC-IV)

The DISC-IV [34] is a structured diagnostic interview and its Dutch version was used to assess the past-year prevalence of the following DSM-IV psychiatric disorders: CD, ODD, attention-deficit/hyperactivity disorder (ADHD), any SUD (i.e., alcohol, marijuana, and/or other drug use disorder), any affective disorder (i.e., depression and dysthymia) and any anxiety disorder (i.e., separation anxiety and post-traumatic stress disorder). Girls with CD were subdivided in childhood-onset (i.e., first symptom prior to 10) and adolescent-onset cases (i.e., first symptom at 10 or later).

#### Quick Big Five (QBF)

The QBF [35] is a Dutch self-report instrument that consists of 30 items that youth are asked to rate on a 7-point Likert scale (1 = “definitely not” to 7 = “very well”). Two of the five scales (five items each) include Agreeableness (*α* = 0.79) and Conscientiousness (*α* = 0.86). Details for the relation between (SF-)ICU scores and the other scales (Extraversion, Emotional Stability, and Openness) are available upon request from the first author.

#### Self-Reported Offending

By means of a Dutch self-report measure [36] youth indicated if they have committed a variety of different criminal acts during their life. Similar to prior studies (e.g., [37]), domain specific scales were created indicating the number of different acts committed within six mutually exclusive categories: Violence (four acts; e.g. using violence or threat of using violence to steal from someone, causing someone injuries in a fight’; *α* = 0.65; *MIC* = 0.15); Property offenses (11 acts; e.g. selling stolen property, burglary; *α* = 0.47; *MIC* = 0.28); Vandalism (six acts, e.g. damaging a car or house; *α* = 0.17; *MIC* = 0.16); Dealing drug (three acts, e.g., selling marijuana; *α* = 0.98; *MIC* = 0.97), and Threats and insults (three acts; e.g., making someone scared through email, threatening someone at school; *α* = 0.62; *MIC* = 0.17).

#### Socio-demographics

Standardized information about age and origin (e.g., Belgian, Moroccan) was assessed by means of a self-report questionnaire designed by the authors.

### Analytic Strategy

Confirmatory factor analysis (CFA) was first used to examine whether the 3-bifactor structure of the ICU provided a good fit to the data using a mean and variance adjusted weighted least squares estimator appropriate for use with ordinal items [38], in Mplus 7.11 [39]. Analyses then examined whether a recently proposed 2-factor model that includes a subset of the original ICU items provided a good fit to the data. Indices used to assess overall model fit included the Chi square (*χ*^2^), comparative fit index (*CFI*), the Tucker-Lewis index (*TLI*) and the root mean square error of approximation (*RMSEA*). With regard to *χ*^2^, a good fit is indicated when *χ*^2^/*df* ≤ 2, whereas *χ*^2^/*df* ≤ 3 is indicative of an acceptable fit [40]. *CFI* and *TLI* values of 0.95 or greater were indicative of good fit and values within the range of 0.90–0.94 indicated acceptable fit. *RMSEA* values less than 0.05 indicated good model fit, while values below 0.08 demonstrated acceptable fit [41]. We next examined the internal consistency of the (SF-)ICU scores. Cronbach’s alpha (*α*) was calculated, with reliability coefficients <0.60 being considered poor, 0.60–0.69 being marginal, 0.70–0.79 being acceptable, 0.80–0.89 being good, and 0.90 being excellent [42]. Because *α* penalizes short scales [43], we also examined the mean corrected item-to-total correlation (*MCITC*) and the mean inter-item correlation (*MIC*) that should be above the conventionally recommended value of 0.30 (*MCITC*) or in the range of 0.15–0.50 (*MIC*) [44, 45]. To test for criterion validity of, the (SF-)ICU scores were correlated with the YPI CU dimension. To test the convergent validity of the ICU scores, the relationship between the (SF-)ICU scores and theoretically meaningful external variables was examined. Odds ratios (*OR*) were calculated to examine the relation between (SF-)ICU scores and categorical dependent variables. Standardized regression coefficients (*ß*) were calculated to examine the relation between the (SF-)ICU scores and continuous dependent variables. Analyses were also carried out that examined the relationship between each (SF-)ICU subscale and the external variables after controlling for the other (SF-)ICU subscales. A *p* value of <0.01 was used as an indicator of statistical significance. To restrict the number of analyses, the *OR* and *ß* were compared to one another based on their value rather than using a formal test.

## Results

### Factor Structure of the ICU and Short-Form ICU

Model fit indices indicated poor fit for the 3-bifactorial model (*χ*^2^ = 723.08, *df* = 228, *χ*^2^/*df* = 3.17, CFI = 0.69, *TLI* = 0.62, *RMSEA* = 0.11). Acceptable fit was demonstrated for the SF-ICU the 2-bifactor model (*χ*^2^ = 83.99, *df* = 42, *χ*^2^/df = 2.00, *CFI* = 0.94, *TLI* = 0.91, *RMSEA* = 0.07). Because current items from the Unemotional subscale may tap the tendency to hide emotions rather than lacking emotions or superficially expressing emotions (e.g., [3]), and because the sole Unemotional item included in the SF-ICU has shown the lowest loading on the Callousness factor [3], it was tested if the fit of the 2-factor model could be improved by removing the only item remaining from the Unemotional subscale. The fit indices improved after this item was removed for the 2-bifactor model (*χ*^2^ = 58.51, *df* = 33, *χ*^2^/df = 1.77, *CFI* = 0.96, *TLI* = 0.94, *RMSEA* = 0.06). From here forward, we used the 2-factor model that excluded this item (i.e., “Does not show emotions”).

### Descriptive Statistics and Reliability Estimates

Reliability estimates and descriptive statistics for the (SF-)ICU are provided in Table 1. The correlations between the ICU total and subscale scores were strong (i.e., Callous-ness: *r* = 0.75; Uncaring: *r* = 0.73; Unemotional: *r* = 0.59; *p*s < .001). The Callousness subscale demonstrated a relatively weak correlation to the Uncaring (*r* = 0.19, *p* < .01) and Unemotional (*r* = 0.19, *p* < .01) subscales, while the Uncaring and Unemotional subscales were moderately intercorrelated (*r* = 0.34, *p* < .001). Using the SF-ICU, the correlation between the SF-total and the SF-Callousness and Uncaring subscales was 0.87 and 0.73 (*p*s < .01), respectively. The correlation between the SF-Callousness and Uncaring subscales was in the low-moderate range (*r* = 0.29, *p* < .001). The ICU and SF-ICU scores were highly correlated (Total: *r* = 0.73; Callousness: *r* = 0.94; Uncaring: *r* = 0.91; *p*s < .001).

**Table 1:** Descriptive statistics and reliability estimates for the Inventory of Callous-Unemotional Traits (ICU)

	Current sample						
	Mean	SD	Min, max	Theo. range	Alpha	MIC	MCITC
*Original ICU*							
Total score	27.73	10.25	3–57	0–72	0.79	0.14	0.34
Callousness subscale	9.42	6.09	0–30	0–33	0.78	0.25	0.43
Uncaring subscale	10.23	5.22	0–21	0–24	0.78	0.32	0.59
Unemotional subscale	8.07	3.18	0–15	0–15	0.52	0.18	0.30
*Short*-*form ICU*							
Total score	9.14	5.88	0–25	0–33	0.76	0.22	0.41
Callousness subscale	4.83	4.22	0–21	0–21	0.72	0.40	0.46
Uncaring subscale	4.31	3.05	0–12	0–12	0.74	0.29	0.51

*Theo.* theoretical, *MIC* mean inter-item correlation, *MCITC* mean corrected-item-to-total correlation

### Criterion Validity

The ICU total scores and the three subscales were significantly related to the CU dimension of the YPI. After controlling for the other two subscales, the Callousness and Uncaring, but not the Unemotional subscale, remained significantly related to YPI CU traits. The SF-ICU total score and the two SF-ICU subscales (whether or not controlling for the other SF-ICU subscale) were also significantly related to YPI CU traits (Table 2).

**Table 2:** Standardized beta coefficients as indicator for the strength of the relation between the Inventory of Callous-Unemotional Traits (ICU) and the Youth Psychopathic Traits Inventory (YPI)

	YPI			
	Callous-Unemotional	Grandiose-manipulative	Impulsive-irresponsible	Total
*Original ICU*				
Total score	.60**	.33**	.36**	.51**
Callousness subscale	.57** (.51**)	.31** (.28**)	.35** (.32**)	.48** (.43**)
Uncaring subscale	.34** (.20*)	.20* (.14)	.23* (.17)	.30** (.20*)
Unemotional subscale	.29** (.13)	.14 (.04)	.12 (.00)	.21* (.06**)
*Short*-*form ICU*				
Total score	.62**	.31**	.33**	.49**
Callousness subscale	.52** (.42**)	.24* (.17)	.30** (.25*)	.41** (.33**)
Uncaring subscale	.48** (.36**)	.27** (.22*)	.23* (.16)	.38** (.29**)

Numbers not between parentheses are Standardized Beta Coefficients from Univariate analyses and can be interpreted as correlation coefficients(<0.30 = weak; 0.30< to <0.50 = moderate; and <0.50 = strong); numbers between parentheses are Standardized Beta Coefficients from multivariate analyses (i.e. including all three ICU subscales simultaneously in the analysis)

* *p* < .01; ** *p* < .001

### Convergent Validity^2^

#### Psychopathic Traits: Grandiose-Manipulative and Impulsive-Irresponsible Traits

The ICU total score was significantly related to YPI GM and II traits. The Callousness and Uncaring subscales, but not the Unemotional subscale, were significantly related to both YPI dimensions. After controlling for the other ICU subscales, only the Callousness subscale remained significantly related to YPI GM and II traits. At the zero-order level, all SF-ICU scores were significantly related to the GM and II dimensions of the YPI. After controlling for the other SF-ICU subscale, SF-Callousness and SF-Uncaring was significantly related to YPI II and YPI GM, respectively.

#### Psychiatric Disorders

Table 3 shows that females with a high ICU total score were more likely to have ODD, CD, and childhood-onset CD (CoCD). Females with a high score on Callousness were more likely to have CD, a finding that remained after controlling for the other two ICU subscales. Females with a high score on Uncaring were more likely to have ODD, CD, and CoCD a finding that remained after controlling for the other two ICU subscales. The Unemotional subscale was not significantly related to any of these disorders. These findings were replicated with the SF-ICU, except that after controlling for the other subscale, the SF Callousness subscale was no longer related to CD.

**Table 3:** Odds ratios as an indicator for the strength of the relation between the Inventory of Callous-Unemotional Traits (ICU) and psychiatric disorders

	ADHD	ODD	CD	CoCD	AoCD	SUD
*Original ICU*						
Total score	1.03	1.05*	1.07**	1.09**	1.01	1.03
Callousness subscale	1.04 (1.03)	1.06 (1.05)	1.09* (1.08*)	1.07 (1.05)	1.03 (1.03)	1.04 (1.03)
Uncaring subscale	1.08 (1.08)	1.10* (1.10*)	1.13** (1.13*)	1.20** (1.18**)	1.00 (1.00)	1.06 (1.05)
Unemotional subscale	1.01 (0.96)	1.04 (0.97)	1.04 (0.96)	1.13 (1.03)	0.96 (0.95)	1.03 (0.98)
*Short*-*form ICU*						
Total score	1.05	1.10*	1.12**	1.12**	1.01	1.06
Callousness subscale	1.05 (1.03)	1.09 (1.06)	1.11* (1.07)	1.08 (1.02)	1.03 (1.04)	1.07 (1.05)
Uncaring subscale	1.11 (1.09)	1.19* (1.16*)	1.23** (1.20*)	1.35** (1.33**)	0.99 (0.98)	1.08 (1.06)

*ADHD* Attention-Deficit/Hyperactivity Disorder, *ODD* Oppositional Defiant Disorder, *CD* Conduct Disorder, *Co* childhood-onset, *Ao* adolescent-onset, *SUD* Substance Use Disorders; numbers not between parentheses are odds ratios from Univariate analyses; numbers between parentheses are odds ratios from multivariable analyses (i.e. including all three ICU subscales simultaneously in the analysis)

* *p* < .01; ** *p* < .001

#### Externalizing Problems

The ICU total score and the Callousness subscale was positively related to rule-breaking behaviour, aggression, and attention problems (Table 4). The Callousness subscale remained positively related to these three variables after controlling for the other two ICU subscales. The Uncaring subscale was positively associated with rule-breaking behaviour and aggression. After controlling for the other two ICU subscales, the relation between the Uncaring subscale and rule-breaking behaviour became non-significant. The Unemotional subscale was not related to externalizing problems. Findings using the SF-ICU total and SF-Callousness and Uncaring subscales were largely consistent with those found using the ICU (see Table 4)

**Table 4:** Standardized beta coefficients as indicator for the strength of the relation between the Inventory of Callous-Unemotional Traits (ICU) and the externalizing problems, self-reported offending and the quick big five personality dimenions

	Rule- breaking	Aggressive behavior	Attention problems	Violent offenses	Theft	Vandalism	Threats Insults	Drug offenses	Agreeableness	Conscien tiousness
*Original ICU*										
Total score	.34**	.43**	.28**	.31**	.30**	.45**	.31**	.17	−.43**	−.39**
Callousness subscale	.31** (.28**)	.35** (.29**)	.28** (.25*)	.21* (.16)	.26** (.22*)	.34** (.28**)	.24* (.19*)	.13 (.10)	−.16 (−.06)	−.15 (−.05)
Uncaring subscale	.24* (.19)	.37** (.32**)	.19 (.15)	.29** (.25*)	.22* (.17)	.36** (.30**)	.24* (.19)	.16 (.14)	−.45** (−.37**)	−.40** (−.32**)
Unemotional subscale	.10 (−.01)	.13 (−.03)	.07 (−.03)	.13 (.02)	.12 (.02)	.19* (.04)	.15 (.05)	.06 (−.01)	−.33** (−.19*)	−32** (−.20*)
*Short*-*form ICU*										
Total score	.31**	.41**	.22*	.32**	.32**	.43**	.32**	.20*	−.34**	−26**
Callousness subscale	.29** (.25*)	.33** (.25*)	.23* (.22*)	.20* (.11)	.25* (.18)	.32** (.22*)	.25** (.19*)	.14 (.09)	−.19* (−.08)	−.13 (−.04)
Uncaring subscale	.21* (.13)	.34** (.27**)	.11 (.04)	.33** (.30**)	.28** (.23*)	.40** (.33**)	.27** (.22*)	.19* (.16)	−.40** (−.37**)	−.33** (−.32**)

Numbers not between parentheses are Standardized Beta Coefficients from Univariate analyses and can be interpreted as correlation coefficients (<0.30 = weak; 0.30< to <0.50 = moderate; and <0.50 = strong); numbers between parentheses are Standardized Beta Coefficients from multivariable analyses (i.e. including all three ICU subscales simultaneously in the analysis)

* *p* < .01; ** *p* < .001

#### Self-Reported Offending

The ICU total score and Callousness and Uncaring subscales were significant positively related to all types of offending, except drug offenses (Table 4). When controlling for the other two ICU subscales, the Callousness subscale was no longer related to violent offenses, while the Uncaring subscale only remained significantly related to violent offenses and vandalism. The Unemotional subscale was only related to vandalism, though this association became non-significant after controlling for the other subscales. The SF-ICU demonstrated similar associations to those seen with the ICU. However, after controlling for the other SF-ICU subscale, the SF-Callousness subscale was no longer significantly related to theft, while the SF-Uncaring subscale showed a significant positive relationship to theft and threats and insults.

#### Big Five Personality

The ICU total score was negatively related to agreeableness and conscientiousness (Table 4). The Callousness subscale was not related to these personality dimensions, while the Uncaring and Unemotional subscale were negatively related to agreeableness and conscientiousness, even after controlling for the other two ICU subscales. These findings were replicated when using the SF-ICU, except that the SF-Callousness subscale was also significantly negatively correlated with agreeableness at the zero-order level.

### Exploring Relations With Internalizing Disorders and Problems

Table 5 shows that none of the ICU and SF-ICU scores were significantly related to affective disorder and withdrawn-depressed feelings. The ICU and SF-ICU total score were negatively related to anxious-depressed feelings and ICU and SF-ICU Uncaring subscale scores were negatively related to anxiety disorder and anxious-depressed feelings. No other significant relations were revealed.

**Table 5:** Odds ratios (OR) and standardized beta coefficients (β) as indicator for the strength of the relation between the Inventory of Callous-Unemotional Traits (ICU) and internalizing disorders and problems

	Affective disorder(s)	Anxiety disorder(s)	Anxious-Depressed	Withdrawn-Depressed
	OR	OR	β	β
*Original ICU*				
Total score	0.97	0.98	−.23*	.08
Callousness subscale	0.97 (0.98)	1.00 (1.01)	−.04 (.01)	.12 (.12)
Uncaring subscale	0.94 (0.95)	0.93 (0.92*)	−.36** (−.38**)	−08 (−.17)
Unemotional subscale	0.95 (0.99)	1.00 (1.05)	−.06 (.07)	.17 (.20)
*Short*-*form ICU*				
Total score	0.95	0.97	−.23*	.02
Callousness subscale	0.96 (0.99)	1.00 (1.03)	−.04 (.08)	.10 (.14)
Uncaring subscale	0.88 (0.89)	0.87* (0.86*)	−.39** (−.42**)	−.09 (−.13)

Numbers not between parentheses are OR and β from Univariate analyses; numbers between parentheses are OR and β from multivariable analyses (i.e. including all three ICU subscales simultaneously in the analysis)

* *p* < .01; ** *p* < .001

## Discussion

This study specifically aimed to examine the factor structure of the (SF)-ICU; associations between (SF-)ICU scores and YPI measured CU traits; and associations between (SF-)ICU scores and theoretically meaningful constructs. Results are discussed first in regards to the ICU, followed by an evaluation of the results as related to the SF-ICU.

### The ICU

Reliability estimates for the ICU are consistent with previous findings which indicate that the internal consistency of the measure is generally good. However, although the three ICU subscales are intended to measure inter-related aspects of the same overarching CU construct, the correlations between these ICU subscales were in the low to low-moderate range. These weak correlations have been reported in studies among community [7, 9] and detained adolescents [10, 46], although the Callousness and Uncaring subscales typically demonstrate a higher correlation than found in the current study (*r* = 0.19). Also in line with prior work [4, 7], the Callousness and Uncaring subscales generally demonstrated higher correlations with conduct problems and antisocial behaviours than they did with each other. Overall, these findings suggest that the ICU subscales may not be tapping into the same overarching CU construct, especially since they have greater overlap with externalizing problems than with each other. For now, it seems prudent to investigate these scales in isolation given their low intercorrelations.

The ICU total score and the ICU Callousness subscale showed strong correlations with the YPI CU dimension. The finding that the ICU subscales generally showed low and non-significant correlations with the YPI GM and II dimensions also questions the extent to which ICU-measured CU traits relate to other psychopathic-traits, at least as measured by the YPI. This poor support for the criterion validity of ICU scores may be restricted to detained females, though future studies are warranted to test this speculation. Prior studies with detained boys [16], and gender-mixed samples of community adolescents [7] and detained adolescents [10], indeed, showed that the strength of the correlations between ICU scores and the CU dimension of other tools were higher or similar to that of the correlations between ICU scores and the other psychopathy dimensions, though not without some exceptions (e.g., [7, 10, 18]).

The ICU total score was positively related to ODD, aggression, rule-breaking behaviour and prior offending, supporting the convergent validity of the ICU total score. However, inspection of the ICU subscales and these external variables demonstrates a somewhat more complicated pattern of findings. For example, the Unemotional subscale was generally not related to aggression and offending. Although this finding converges with previous studies [5], it is at odds with theoretical conceptualizations of psychopathic-like traits being related to aggression and criminal behavior, and again calls into question the utility of the Unemotional subscale as an index of CU traits. Finally, and against expectations, ICU scores were not related to substance use disorders.

CU traits are considered to identify an important psychopathic-like subgroup of youth with CoCD [23]. Therefore, one would expect CU traits to be positively related to CD, and that CU traits would be more strongly related to CoCD than AoCD [25]. We showed that ICU scores, except the Unemotional subscale, were positively related to CD, and that the ICU total score and Uncaring subscale were significantly related to CoCD, but not to AoCD. These results provide some support for using CU traits to identify a particularly high-risk group of CD girls, and for current attempts to integrate CU traits into the diagnosis of CD, especially childhood-onset CD.

### The SF-ICU

Using a slightly modified 2-factor model, we were able to replicate the 2-factor structure proposed by Hawes [3]. Also consistent with the initial validation of the factor structure [3], strongest support for this model was found after excluding the sole item from the Unemotional subscale. The SF-ICU showed good model fit and appeared to be internally consistent according to various reliability estimates. This is particularly encouraging if one considers that the SF-Uncaring subscale only includes four items. Finally, using the SF-ICU also improved the criterion validity of the Uncaring subscale as measured by its association to the YPI CU dimension. In sum, the SF-ICU may resolve several psychometric problems that have been reported previously for the ICU. Yet, it may be premature to completely eliminate the Unemotional subscale in the ICU. Indeed, the SF-ICU version identified by Hawes (2014) included an item from the Unemotional subscale, whereas the present study showed that the Unemotional subscale was associated with agreeableness and conscientiousness in a meaningful way. Therefore, future studies with a focus on improving rather than eliminating the Unemotional items of the ICU are also warranted.

Some issues remain and need to be addressed in future SF-ICU studies. First, the correlation between the Callousness and Uncaring increased from 0.19 to 0.29 when the SF-subscales were used. Yet, this correlation is still low for two subscales that are assumed to measure the same construct, and much lower than the correlation of the SF-ICU parent-version scales [3]. It is possible that the strength of the correlation between these ICU subscales mainly depends upon the informant used to assess CU traits. The finding that both scales were more strongly correlated when using the ICU parent version than when using the ICU self-report version (*r*s = 0. 45 and 0.36) supports this suggestion [10]. It is also possible that the attenuated correlations between the Uncaring and Callous factors are due, at least in part, to the items from each of these factors discriminating the overarching CU construct at opposite ends of the continuum. This again may point toward potential concerns of a method factor stemming from differences in positively and negatively worded items that comprise these constructs or may indicate that further refinement of these factors is needed to ascertain that they are each tapping into the overarching CU construct as intended. Second, although most relations between the ICU subscales and these variables were replicated when using the SF-Callousness and Uncaring subscales, both SF-subscales are still less strongly or only equally related to each other than to several indices of antisocial behaviour. Future research on this topic is needed, particularly because prior work with the SF-ICU reported findings that do not converge with the current study’s finding. For example, whereas Hawes (2014) showed that, after controlling for the other SF subscale, only the SF-Callousness subscale was positively related to ODD and CD, the present study showed that only the Uncaring subscale was positively related to these outcomes.

Third, although the SF-ICU score showed a negative relation with anxiety, it is not yet clear how consistently SF-ICU scores relate to internalizing problems, particularly because both positive associations with SF-ICU scores have been revealed as well [3, 12]. Yet, mixed findings between CU traits and internalizing problems have been revealed with other tools, even within the same sample (e.g., [30]), and emphasize the importance of further examining CU traits in relation to internalizing problems.

### Implications

There are a number of implications for the assessment of CU traits with the (SF-)ICU in detained female adolescents. First, CU traits have been considered an important construct to assess in detained adolescents [18]. The psychometric problems reported here and elsewhere (e.g., [12]), and the contrasting recommendations to merely use the total ICU score [20] or to only use the ICU subscale scores (present study), suggest that researchers and clinicians should not only use this tool to assess CU traits.

Second, it has been argued that the ICU Unemotional items do not appear to operate as intended in the nomological network of CU traits, and therefore may not be useful clinically or conceptually [12]. Yet, various studies ranging from behavioural over genetic to brain imaging studies relied on the ICU total score (the Unemotional items included), underscoring the importance to replicate findings from these studies using the SF-ICU or other measures of CU traits.

Third, although the SF-ICU resolved various problems that have been reported previously for the ICU, the SF-ICU does not include items that have been selected to assess the DSM-5 specifier criterion ‘Concerned about performance at school, work, or in other important activities” (e.g., [23]). In addition, having only one item [3] or no item (this study) that assesses unemotionality also implies that the SF-ICU does not allow to comprehensively assess the DSM-5 specifier criterion ‘Shallow and Deficient Affect’. So, while the ICU content was designed to provide a continuous measure of CU traits, similar to how they are operationalized for the *DSM*-*5* specifier [20], the items selected for SF-ICU restrict the possibility to assess CU traits as defined by the DSM-5 specifier.

Fourth, if so few Unemotional items are included in the SF-ICU, it may be beneficial to start referring to Callous-Uncaring rather than Callous-Unemotional traits, when using this measure.

### Study Limitations

The current study has several strengths, including the largest sample of detained female adolescents available to date on the psychometric properties of the ICU; and the use of well-validated measures to assess the criterion, and convergent validity of the (SF-)ICU scores. As always, the findings must be interpreted in the context of various limitations. The use of a severe antisocial and behaviour disordered female only sample does preclude direct comparisons among genders, and implies that this is the only population that an inference can be drawn upon. Our sole reliance on self-report could be considered as a limitation. However, studies that rely on information supplied by a single informant often demonstrate method variance which can lead to inflated relations among study variables. From this point of view, the poor to moderate correlations between the ICU and variables of interest are particularly worrisome. Due to a difference in the number of items that need to be reverse scored between the Dutch and the English ICU, studies are also warranted to see if the SF-factor model can be replicated in other countries. Finally, future studies are needed to test if ICU scores remain significantly related after removing its shared variance with the other dimensions of the psychopathy construct, an issue that was beyond the scope of this paper [47].

### Summary

Studies that rely on the ICU must use the total score with great caution given the inconsistent relation between its subscales and external variables. This is particularly true for the Unemotional subscale. It is recommended that investigators who wish to use the total score in their research also conduct analyses using the subscale scores, even if these are supplementary in nature. This study also showed that the SF-ICU may help to improve the factor structure of the ICU. Yet, future studies are needed to test whether the present study findings can be generalized to other samples of boys and girls.

## Footnotes

1 For brevity, we will use “(SF-)ICU” when referring to both the ICU and the SF-ICU. So, SF-ICU is only used when we specifically refer to the SF-ICU.

2 Descriptive information for all variables of interest can be retrieved in a previous publication (30).
